# ASO Author Reflections: R0 Resection Should Not Be Dismissed in Node-Positive Pancreatic Ductal Adenocarcinoma

**DOI:** 10.1245/s10434-026-20047-y

**Published:** 2026-07-24

**Authors:** A. M. Bonomi, S. Granieri, R. M. Montorsi, E. Gjoni, O. R. Busch, A.A. Vidal-Itriago, J.I. Erdmann, M. G. Besselink

**Affiliations:** 1 ASST Brianza – Vimercate Hospital, Department of Surgery, Vimercate, Italy; 2 Department of Surgery, Amsterdam UMC, location University of Amsterdam, Amsterdam, the Netherlands; 3 Cancer Center Amsterdam, Amsterdam, the Netherlands; 4 Acute care and Trauma Surgery, ASST GOM Niguarda, Milan, Italy; 5 Medical Library, Vrije Universiteit Amsterdam, Amsterdam, The Netherlands

## Past

For decades, pancreatic surgeons have been taught that an R0 resection should be pursued almost at any cost, with the implicit belief that wider and more radical procedures would automatically translate into meaningful survival gains. This paradigm has been reinforced by historical series showing clear survival differences between R0 and R1 resections in unselected pancreatic ductal adenocarcinoma (PDAC) cohorts and by guidelines that place margin-negative resection at the center of curative-intent surgery. As a result, surgical strategy, intraoperative decision-making, and even referral patterns have often been driven by the imperative to “clear the margin,” sometimes with limited attention to tumor biology, patient fitness, and standardization of pathological assessment.

## Present

More recently, data in node-positive PDAC have challenged the dogma that margin status always retains major prognostic weight, leading some to suggest that R1 resection might be acceptable or even unavoidable in advanced disease stages.^1^ The results of our systematic review and meta-analysis of resected node-positive pancreatic ductal adenocarcinoma, limited to patients undergoing upfront surgery (somewhat unexpectedly, only a small minority had received neoadjuvant therapy, and data were insufficient to allow subgroup analysis), show that R1 resection still carries a significantly increased risk of death compared with R0, even when margins are assessed with Royal College of Pathologists (RCP)–based protocols.^2^ Importantly, we observed wide variability in reported R0 and R1 rates across studies, highlighting that failure to adopt and/or apply standardized inking, axial slicing, and the 1-mm rule can misclassify R1 resections as R0 and thereby dilute or distort the true prognostic impact of true R0 resections. Thus, in the current treatment era, the interpretation of R0 and R1 must be inseparable from how margins are processed and reported, making standardized pathology and distinction between *surgical R1* (i.e., R1 = tumor at margin ink) and *pathological R1* (i.e., R1 = tumor within 1 mm of margin) prerequisites for any meaningful discussion of oncologic quality.

## Future

Looking ahead, our findings should not be extrapolated uncritically to all patients, especially those receiving modern neoadjuvant and adjuvant regimens. Emerging studies suggest that R1 may “lose” prognostic significance after adjuvant FOLFIRINOX; ^3^ yet, as highlighted by recent high-quality series of extended resections after intensive FOLFIRINOX, R1 can still be a powerful negative prognostic factor when surgery and pathology are truly optimized, ^4^ raising the concern that dismissing margin status may be misleading and even dangerous. Future research should therefore adopt rigorous, RCP-compliant principles and a harmonized margin assessment methodology as a non-negotiable standard, so that trials comparing surgical strategies, systemic regimens, or neoadjuvant versus upfront approaches do not conflate biological differences with variability in pathological technique, which still requires definitive interobserver and interinstitutional standardization. ^5^ Rather than using imperfect or non-standardized data to justify suboptimal resections, the next step should be to clarify when an R1 resection—assuming optimal surgical technique, standardized back-table inking, and expert pathology—is acceptable compared with continuing or intensifying systemic therapy, with the ultimate goal of achieving the best possible outcome for each patient, including the possibility that, for some, not proceeding to a Whipple or an “inevitable” R1 resection may be the most appropriate choice.
